# Dr. Levi C. Taylor

**Published:** 1922-07

**Authors:** 


Dr. Levi C. Taylor

Dr. Levi Colby Taylor died February 8, 1922, at his home, Hartford, Conn., of carcinoma of the intestines, in his eighty-first year.

Dr. Taylor was born in Lempster, N. H., December 12, 1841, the son of Erastus Day and Mary (Colby) Taylor. He received his early education in the district schools of Lempster and later at the Academy in Henniker, N. H.

Dr. Taylor early became interested in dentistry and entered the office of Dr. George Bowers of Springfield, Vt., where he served an apprenticeship of several years. In 1868 he established himself in the practice of dentistry in Holyoke, Mass., where he remained until 1875. In 1875 he removed to Hartford, Conn., where he became associated in practice with Dr. John M. Riggs.

Dr. Taylor was a practitioner of the old school and a man who exerted every effort to keep in close touch with the progressive stages of dentistry, and with marked success. Entering the practice of dentistry before the establishment of the dental laws regulating the practice of dentistry and before professional education was generally available, Dr. Taylor, through characteristics which he inherited from the Puritan stock of the Massachusetts Bay colony, became one of the leading practitioners of his community. He was one of the leading exponents of the practice of preventive dentistry and for many years preached and practiced the doctrine of prophylactic dentistry. Somewhat handicapped by defective hearing, he nevertheless was a constant attendant at almost all the dental society meetings of New England. He was one of the early members of the old Connecticut Valley Dental Society, which later became the Northeastern Dental Association, whose meetings he attended almost without exception for forty years. He was a constant attendant at the meetings of the Connecticut State Dental Society and took an active part in all of the activities of the association.

Dr. Taylor was lecturer on Oral Prophylaxis at the New York College of Dental and Oral Surgery, New York, from 1892 to 1904. He was at one time president of the Connecticut Valley Dental Association and was the first president of the Hartford Dental Society; he was also honorary member of the Massachusetts State Dental Society, the New Hampshire Dental Association, and the Institute of Stomatology of New York. He had practiced his profession continuously for fifty-five years up to within a week of the time of his death.

Dr. Taylor was married in December, 1874, to Miss Nellie Thayer of Peterboro, N. H. He is survived by his widow, a daughter, Dr. Maude W. Taylor, of Hartford, and a grand-daughter, Betsy Allen Taylor, of Rockhill, Conn. The interment took place February 11, 1922, in Spring Grove Cemetery, Hartford, Conn.—*Cosmos.*

